# Linguistic neutrosophic power Muirhead mean operators for safety evaluation of mines

**DOI:** 10.1371/journal.pone.0224090

**Published:** 2019-10-24

**Authors:** Suizhi Luo, Weizhang Liang, Guoyan Zhao

**Affiliations:** 1 College of Systems Engineering, National University of Defense Technology, Changsha, Hunan, China; 2 School of Resources and Safety Engineering, Central South University, Changsha, Hunan, China; Vietnam National University, VIET NAM

## Abstract

Safety is the fundamental guarantee for the sustainable development of mining enterprises. As the safety evaluation of mines is a complex system engineering project, consistent and inconsistent, even hesitant evaluation information may be contained simultaneously. Linguistic neutrosophic numbers (LNNs), as the extensions of linguistic terms, are effective means to entirely and qualitatively convey such evaluation information with three independent linguistic membership functions. The aim of our work is to investigate several mean operators so that the safety evaluation issues of mines are addressed under linguistic neutrosophic environment. During the safety evaluation process of mines, many influence factors should be considered, and some of them may interact with each other. To this end, the Muirhead mean (MM) operators are adopted as they are powerful tools to deal with such situation. On the other hand, to diminish the impacts of irrational data provided by evaluators, the power average (PA) operators are under consideration. Thus, with the combination of MM and PA, the power MM operators and weighted power MM operators are proposed to aggregate linguistic neutrosophic information. Meanwhile, some key points and special cases are studied. The advantages of these operators are that not only the interrelations among any number of inputs can be reflected, but also the effects of unreasonable information can be reduced. Thereafter, a new linguistic neutrosophic ranking technique based on these operators is developed to evaluate the mine safety. Moreover, in-depth discussions are made to show the robust and flexible abilities of our method. Results manifest that the proposed method is successful in dealing with mine safety evaluation issues within linguistic neutrosophic circumstances.

## Introduction

Mineral resources are important raw materials for other downstream industries, which play a fundamental role in economic development [1,2]. Safety production is a prerequisite for the exploitation of mineral resources, and is an important guarantee for the sustainable development of mining enterprises. Nevertheless, mining is full of high risks due to the industry particularity. Although safety issues are getting more and more attentions, many dangers still exist in the mining process. Every year, plenty of casualties are caused by mining accidents around the world, especially in developing countries [3,4]. Only take coal mines in China as an example, the number of deaths has reached at least 275 in 2018 [5]. To guarantee the safety of miners and take effective control measures in advance, it is significant to adopt effective approach to conduct mine safety evaluation.

Mine safety evaluation can be regarded as an decision making process with the purpose of picking out the safest mine or ranking mines based on their safety performance. Due to the complexity of mine system, the mine safety is affected by many criteria, such as the individual, environmental and managerial factors. Accordingly, many multi-criteria decision making (MCDM) methods have been used to solve mine safety evaluation problems, such as the analytic hierarchy process (AHP) [3], ordered weighted aggregation operator [6], and Frank Heronian mean (HM) operator [7]. In addition, as numerous uncertainties are contained in the working environment of mines, decision makers (DMs) often have a vague understanding when conducting safety assessment. In this case, they are accustomed to using linguistic phrases (i.e., “very good”, “good” and “bad”) instead of numerical values [8].

Zadeh [9] firstly put forward the notion of linguistic variables to represent linguistic evaluation information. Thereafter, plenty of decision making methods based on linguistic variables have been developed [10,11,12]. On the other hand, considering the vagueness of human cognitions, linguistic terms have been combined with various fuzzy sets to express more uncertain information [13,14,15]. For example, Chen et al. [16] defined the linguistic intuitionistic fuzzy numbers (LIFNs) based on linguistic terms and intuitionistic fuzzy numbers (IFNs). In IFNs, two crisp numbers are respectively adopted to describe the membership and non-membership functions [17,18]. In LIFNs, both the membership and non-membership degrees are linguistic values, rather than crisp numbers. As a result, the incomplete evaluation information can be qualitatively described. However, for both IFNs and LIFNs, there is still an obvious limitation, that is: (1) In a group decision making process, inconsistent results are likely to be produced among several DMs. Another situation is that people may be hesitant about their evaluations when facing with complex objectives. Nevertheless, IFNs and LIFNs cannot address such situations because they don’t contain indeterminate or inconsistent linguistic data.

To conquer the limitation (1) of IFNs, Smarandache [19] first proposed the notion of neutrosophic sets (NSs). When there is only an element in NSs, it is reduced as a single-valued neutrosophic number (SVNN) [20]. Three membership functions (namely, the truth, hesitance and falsity membership degrees) are within a SVNN [21,22]. As a result, all the consistent, hesitant and inconsistent information of DMs can be contained in a SVNN. From then on, various decision making models related to NSs have been presented [23,24,25,26]. Besides, considering the advantages of NSs, they have been extended with some other fuzzy numbers to deal with complex real problems [27,28,29]. For example, Ji et al. [30] proposed a combined neutrosophic linguistic approach to pick out ideal providers; Liu et al. [31] put forward the Dombi power HM operators under 2-tuple linguistic neutrosophic environment; Abdel-Basset et al. [32] used type-2 neutrosophic number to describe linguistic phrases in the decision process; Wang et al. [33] extended the Muirhead mean operators with neutrosophic 2-tuple linguistic information; Dat et al. [34] discussed the interval complex neutrosophic sets within linguistic decision circumstances.

Borrowed the idea of single-valued neutrosophic numbers (SVNNs), the concept of linguistic neutrosophic numbers (LNNs) [35] was raised to overcome this drawback (1) of LIFNs. They extended linguistic terms with SVNNs. This combination can make full use of the advantages of linguistic variables and neutrosophic sets. Three autonomous linguistic membership degrees exist in LNNs so as to comprehensively describe qualitative evaluation information [36]. Consequently, many researchers show great interest in solving decision making problems under linguistic neutrosophic environments [37,38,39]. For example, Shi and Ye [40] defined the cosine measure of LNNs to settle MCDM issues; Liang et al. [41] evaluated the investment risk of metallic mines by using an improved technique for order performance by similarity to ideal solution (TOPSIS) approach within linguistic neutrosophic circumstances; Pamučar et al. [42] selected the best power-generation technique with an extended combinative distance-based assessment (CODAS) model based on LNNs; Liang et al. [43] chose a satisfactory mining method with a linguistic neutrosophic multi-objective optimization by ratio analysis plus the full multiplicative form (MULTIMOORA) method. Moreover, some extensions of LNNs have been studied in existent literature [44,45]. Recently, Liu and You [46] defined a novel distance measure and the bidirectional projection measure of LNNs; Li et al. [47] extended the evaluation based on distance from average solution (EDAS) technique to LNNs for selecting the optimal property management company; Wang et al. [48] combined lingusitic neutrosophic information with the visekriterijumska optimizacija i kom-promisno resenje (VIKOR) method to pick out a suitable fault handling point.

Besides, information aggregation operators are another basic and powerful decision making techniques [49,50]. Consequently, many aggregation operators related to LNNs are also presented. For instance, Fang and Ye [35] described the basic mean operators of LNNs; Garg and Nancy [51] considered the priority relations of linguistic neutrosophic information. However, there are two noticeable shortcomings of these existing operators: (2) There is a hypothesis: All criteria have no relevance with each other. Clearly, it is uncommon in real life; (3) the correlations among inputs are not taken into considerations at all.

To conquer the weaknesses of (2) and (3), some researchers have explored new linguistic neutrosophic aggregation operators. For example, considering that Bonferroni mean operators [52,53] contain the relationships of inputs, Fan et al. [54] proposed the linguistic neutrosophic weighted Bonferroni mean (LNWBM) and linguistic neutrosophic weighted geometric Bonferroni mean (LNWGBM) operators; Wang and Liu [55] generalized the partitioned Bonferroni mean operators within linguistic neutrosophic conditions; Liang et al. [43] proposed several HM operators to do with LNNs. They abandoned the inputs independency assumption and took the relations of arguments into account. Yet, there is still a deficiency in these operators: (4) Even though they regard the inputs are dependent, the interrelationships can only be reflected between two arguments. That is, these operators are useless in the situation where there are more than two inputs are interrelated.

Subsequently, for overcoming this flaw (4), scholars tried to investigate other useful aggregation operators to capture more correlations. For instance, Liu and You [56] combined the weighted Hamy mean operators with linguistic neutrosophic information; Liang et al. [57] extended the Hamacher aggregation operators with LNNs to obtain aggregated results. Except them, another renowned aggregation operators are the family of Muirhead mean (MM) operators [58]. They are powerful and flexible in dealing with correlations among any number of inputs. The largest highlight of these operators is that they can perform diverse functions by allocating different values to the parameter vector according to different conditions. In this sense, some of the mentioned-above operators, such as the basic mean operators and Bonferroni mean operators, can be regarded as the special cases of MM operators. Because the classical MM operators can only treat with crisp numbers, they have been modified with various fuzzy sets to resolve fuzzy decision making methods [59,60,61]. Yet, the imperfections are: (5) As far as we know, the MM operators have not been integrated with LNNs until now; (6) the bad effects of unreasonable inputs on the final aggregated values are ignored.

To surmount the disadvantage of (6), the idea of power average (PA) operators was put forward by Yager [62]. The PA operators have a great performance in eliminating the influence of awkward information provided by DMs [63]. Different with prioritized operators, PA operators allow inputs to support each other in the process of aggregation by defining support degrees (instead of prioritization relationships) [64]. Since then, the PA operators have been either improved with fuzzy extensions to dispose decision making issues under dissimilar settings, or combined with other operators to achieve more goals. For instance, Liu et al. [65] integrated the PA operators with HM operators to aggregate linguistic neutrosophic information. Particularly, the PA operators have been combined with MM operators under many fuzzy environments [66,67]. However, a defect is that: (7) Their integration has not been studied in the linguistic neutrosophic situation. To overcome the limitations of (5) and (7), this study takes this idea for reference and aims to recommend several power Muirhead mean (PMM) operators for LNNs to better resolve complex decision making problems.

In summary, the main motivations of this study are three-fold. First, in the safety evaluation process of mines, consistent, hesitant and inconsistent information may be included in a decision making group at the same time. LNNs are suitable for describing such information with three independent linguistic membership degrees. Second, due to the complexity of objectives or the limitation of DMs’ knowledge, unreasonable data may be provided by DMs. In this case, the PA operators can be adopted to reduce the impacts of these irrational values. Third, as some mine safety evaluation criteria has interactions with each other, proper techniques should be used to capture these relationships. Thus, the objective of our work is to assess the safety of mines through integrating PA with MM operators under linguistic neutrosophic environment.

The key novelties and contributions are:

First, the fuzzy assessment information of the mine safety is expressed with LNNs, so that the disadvantage of (1) is overcome and the preferences or opinions of DMs can be fully conveyed with three independent linguistic membership degrees.

Second, the linguistic neutrosophic power Muirhead mean (LNPMM) and weighted linguistic neutrosophic power Muirhead mean (WLNPMM) operators are suggested to aggregate evaluation information under linguistic neutrosophic environment. Besides, some important properties are certified and special cases are discussed. As a result, the limitations of (2)-(7) can be all surmounted.

Third, a new framework on the basis of these operators is established to solve multi-criteria evaluation problems within linguistic neutrosophic circumstances. An example of assessing safety status of gold mines is provided to explain the utilization of the new method. In addition, its flexibility and superiority are certified after thorough discussions.

The rest of this study is: Section 2 briefly introduces related knowledge of LNNs and PMM operators. In Section 3, the LNPMM and WLNPMM operators, are recommended to aggregate LNNs. After that, a novel approach with these two operators is proposed in Section 4. Next, an illustration instance of safety evaluation of mines is provided to display the application of the presented method in Section 5. At the same time, sensitivity analyses and comparison analyses are conducted in Section 6 to show the features and highlights of this method. Some main conclusions are provided in the end.

## Basic knowledge

Some preliminaries are provided in this section to advance the following studies.

### Linguistic neutrosophic numbers

**Definition 1.** [68] Let ${\bar{a}}_{i}$ (*i* = 0,1,…,2*b*) be a linguistic phrase, then a collection of ${\bar{a}}_{i}$ is regarded as a disconnected linguistic term set $\bar{A}=\left\{{\bar{a}}_{i}|i=0,1,\ldots ,2b\right\}$. If *A* = {*a*_*i*_|*i*∈[0,2*c*]}, it is a continuous linguistic term set.

For two arbitrary linguistic phrases *a*_*i*_ (*i*∈[0,2*c*]) and *a*_*j*_ (*j*∈[0,2*c*]) in *A*, the basic operations contain: *a*_*i*_⊕*a*_*j*_ = *a*_*i*+*j*_ and ∂*a*_*i*_ = *a*_∂*i*_ (∂>0).

Besides, the preference relations between two linguistic phrases are: (1) *a*_*i*_≻*a*_*j*_ if *i*>*j*; (2) *a*_*i~*_*a*_*j*_ if *i* = *j*; (3) *a*_*i*_≺*a*_*j*_ if *i*<*j*.

**Definition 2.** [35] Suppose *A* = {*a*_*i*_|*i*∈[0,2*c*]} is a linguistic term set, then three linguistic membership functions (namely, the linguistic true membership degree *a*_*T*_∈*A*, the linguistic indeterminate membership degree *a*_*I*_∈*A*, and the linguistic false membership degree *a*_*F*_∈*A*) are composed of a linguistic neutrosophic number (LNN), denoted as *α* = (*a*_*T*_,*a*_*I*_,*a*_*F*_).

**Definition 3.** [35] Given two LNNs $\alpha_{1}=(a_{T_{1}},a_{I_{1}},a_{F_{1}})$ and $\alpha_{2}=(a_{T_{2}},a_{I_{2}},a_{F_{2}})$, a linguistic term set *A* = {*a*_*i*_|*i*∈[0,2*c*]}, and ∂>0, their operational rules are

1. $\alpha_{1}⊕\alpha_{2}=(a_{T_{1}},a_{I_{1}},a_{F_{1}})⊕(a_{T_{2}},a_{I_{2}},a_{F_{2}})=(a_{T_{1}+T_{2}-\frac{T_{1}T_{2}}{2c}},a_{\frac{I_{1}I_{2}}{2c}},a_{\frac{F_{1}F_{2}}{2c}})$;
2. $\alpha_{1}⊗\alpha_{2}=(a_{T_{1}},a_{I_{1}},a_{F_{1}})⊗(a_{T_{2}},a_{I_{2}},a_{F_{2}})=(a_{\frac{{}_{T_{1}T_{2}}}{2c}},a_{I_{1}+I_{2}-\frac{I_{1}I_{2}}{2c}},a_{F_{1}+F_{2}-\frac{F_{1}F_{2}}{2c}})$;
3. $\partial \alpha_{1}=\partial (a_{T_{1}},a_{I_{1}},a_{F_{1}})=(a_{2c-2c{\left(1-\frac{T_{1}}{2c}\right)}^{\partial }},a_{2c{\left(\frac{I_{1}}{2c}\right)}^{\partial }},a_{2c{\left(\frac{F_{1}}{2c}\right)}^{\partial }})$;
4. $\alpha_{1}{}^{\partial }={\left(a_{T_{1}},a_{I_{1}},a_{F_{1}}\right)}^{\partial }=(a_{2c{\left(\frac{T_{1}}{2c}\right)}^{\partial }},a_{2c-2c{\left(1‐\frac{I_{1}}{2c}\right)}^{\partial }},a_{2c-2c{\left(1‐\frac{F_{1}}{2c}\right)}^{\partial }})$.

**Definition 4.** [43] Let *α* = (*a*_*T*_,*a*_*I*_,*a*_*F*_) be an LNN, *T*, *I* and *F* are respectively the subscripts of *a*_*T*_∈*A*, *a*_*I*_∈*A* and *a*_*F*_∈*A*, then its score function *B*(*α*) and accuracy function *C*(*α*) are

$$B(\alpha )=\frac{4c+T-I-F}{6c},$$ (1)

$$C(\alpha )=\frac{T-F}{2c}.$$ (2)

**Definition 5.** [43] For two arbitrary LNNs $\alpha_{1}=(a_{T_{1}},a_{I_{1}},a_{F_{1}})$ and $\alpha_{2}=(a_{T_{2}},a_{I_{2}},a_{F_{2}})$, their preference relations are

1. if *B*(*α*_1_)>*B*(*α*_2_), then *α*_1_≻*α*_2_;
2. if *B*(*α*_1_) = *B*(*α*_2_) and *C*(*α*_1_)>*C*(*α*_2_), then *α*_1_≻*α*_2_;
3. if *B*(*α*_1_) = *B*(*α*_2_) and *C*(*α*_1_) = *C*(*α*_2_), then *α*_1_~*α*_2_.

**Definition 6.** [41] If $\alpha_{1}=(a_{T_{1}},a_{I_{1}},a_{F_{1}})$ and $\alpha_{2}=(a_{T_{2}},a_{I_{2}},a_{F_{2}})$ are two LNNs, then their distance can be defined as

$$L(\alpha_{1},\alpha_{2})={\left(\frac{1}{3}\cdot \frac{1}{2c}(|T_{1}-T_{2}{|}^{\lambda }+|I_{1}-I_{2}{|}^{\lambda }+|F_{1}-F_{2}{|}^{\lambda })\right)}^{\frac{1}{\lambda }}(\lambda >0).$$ (3)

When *λ* = 1, the equation is reduced to the Hamming distance $L_{H}(\alpha_{1},\alpha_{2})=\frac{1}{3}\cdot \frac{1}{2c}(|T_{1}-T_{2}|+|I_{1}-I_{2}|+|F_{1}-F_{2}|)$; when *λ* = 2, the equation is reduced to the Euclidean distance $L_{E}(\alpha_{1},\alpha_{2})={\left(\frac{1}{3}\cdot \frac{1}{2c}(|T_{1}-T_{2}{|}^{2}+|I_{1}-I_{2}{|}^{2}+|F_{1}-F_{2}{|}^{2})\right)}^{\frac{1}{2}}$.

### Power Muirhead mean operators

**Definition 7.** [58] Assume *β*_*i*_ (*i* = 1,2,…,*n*) is a group of real numbers, *D* = (*d*_1_,*d*_2_,…,*d*_*n*_)∈*R*^*n*^ is a vector of parameters, *σ*(*j*) (*j* = 1,2,…,*n*) is an arrangement of *i* (*i* = 1,2,…,*n*), and *E*_*n*_ is a set of all possible arrangements, then the MM operators are

$$MM^{D}(\beta_{1},\beta_{2},\ldots ,\beta_{n})={\left(\frac{1}{n!}\sum_{\sigma \in E_{n}}\prod_{j=1}^{n}\beta_{\sigma (j)}^{d_{j}}\right)}^{\frac{1}{\sum_{j=1}^{n}d_{j}}}.$$ (4)

**Definition 8.** [62] Assume *β*_*i*_ (*i* = 1,2,…,*n*) is a set of crisp numbers, $F(\beta_{i})=\sum_{j=1,j\neq i}^{n}G(\beta_{i},\beta_{j})$, and 0≤*G*(*β*_*i*_,*β*_*j*_)≤1 is the support of *β*_*i*_ to *β*_*j*_, then the PA operators are defined as

$$PA(\beta_{1},\beta_{2},\ldots ,\beta_{n})=\sum_{i=1}^{n}\left(\frac{(1+F(\beta_{i}))\beta_{i}}{\sum_{j=1}^{n}\left(1+F(\beta_{j})\right)}\right).$$ (5)

Note that: the support *G*(*β*_*i*_,*β*_*j*_) = *G*(*β*_*j*_,*β*_*i*_); and if *H*(*β*_*i*_,*β*_*j*_)<*H*(*β*_*i*_,*β*_*e*_), then *G*(*β*_*i*_,*β*_*j*_)<*G*(*β*_*i*_,*β*_*e*_), where *H*(*β*_*i*_,*β*_*j*_) is the distance between *β*_*i*_ and *β*_*j*_.

**Definition 9.** [66] If *β*_*i*_ (*i* = 1,2,…,*n*) is a set of crisp numbers, *σ*(*i*) (*i* = 1,2,…,*n*) is any permutation of *i* (*i* = 1,2,…,*n*), *D* = (*d*_1_,*d*_2_,…,*d*_*n*_)∈*R*^*n*^ is a vector of parameters, *E*_*n*_ is a set of all possible permutations, $F(\beta_{i})=\sum_{j=1,j\neq i}^{n}G(\beta_{i},\beta_{j})$ and *G*(*β*_*i*_,*β*_*j*_)∈[0,1] is the support of *β*_*i*_ to *β*_*j*_, then the PMM operators are defined as

$$PMM^{D}(\beta_{1},\beta_{2},\ldots ,\beta_{n})={\left(\frac{1}{n!}\sum_{\sigma \in E_{n}}\prod_{i=1}^{n}{\left(\frac{n(1+F(\beta_{\sigma (i)}))\beta_{\sigma (i)}}{\sum_{j=1}^{n}\left(1+F(\beta_{j})\right)}\right)}^{d_{i}}\right)}^{\frac{1}{\sum_{i=1}^{n}d_{i}}}.$$ (6)

## Some linguistic neutrosophic power Muirhead mean operators

In this section, the PMM operators are extended under linguistic neutrosophic environment. As a result, the LNPMM and WLNPMM operators are put forward to aggregate linguistic neutrosophic information. The largest advantage of these operators is: They could capture the relationships among any number of inputs, at the same time, the influence of unreasonable information can be diminished.

### Linguistic neutrosophic power Muirhead mean operator

**Definition 10.** If *α*_*i*_ (*i* = 1,2,…,*m*) is a group of LNNs, *K* = (*k*_1_,*k*_2_,…,*k*_*m*_)∈*R*^*m*^ is a vector of parameters, *σ*(*i*) is any permutation of (*i* = 1,2,…,*m*), *E*_*m*_ is a set of all possible permutations, $F(\alpha_{i})=\sum_{j=1,j\neq i}^{m}G(\alpha_{i},\alpha_{j})$ and *G*(*α*_*i*_,*α*_*j*_) = 1−*L*(*α*_*i*_,*α*_*j*_)∈[0,1] is the support for *α*_*i*_ and *α*_*j*_, $0\leq w_{i}=\frac{\left(1+F(\alpha_{\sigma (i)})\right)}{\sum_{j=1}^{m}\left(1+F(\alpha_{j})\right)}\leq 1$, and *w*_1_+*w*_2_+⋯+*w*_*m*_ = 1, then the LNPMM operator is

$$LNPMM^{K}(\alpha_{1},\alpha_{2},\ldots ,\alpha_{m})={\left(\frac{1}{m!}\sum_{\sigma \in E_{m}}\prod_{i=1}^{m}{\left(mw_{i}\alpha_{\sigma (i)}\right)}^{k_{i}}\right)}^{\frac{1}{\sum_{i=1}^{m}k_{i}}}.$$ (7)

**Theorem 1.** Suppose $\alpha_{i}=(a_{T_{i}},a_{I_{i}},a_{F_{i}})$ (*i* = 1,2,…,*m*) is a group of LNNs, then the result based on Eq (7) is still an LNN, and

$$\begin{array}{l} LNPMM^{K}(\alpha_{1},\alpha_{2},\ldots ,\alpha_{m})=\\ \left(a_{2c{\left(1-{\left(\prod_{\sigma \in E_{m}}\left(1-\prod_{i=1}^{m}{\left(1-{\left(1-\frac{T_{\sigma (i)}}{2c}\right)}^{mw_{i}}\right)}^{k_{i}}\right)\right)}^{\frac{1}{m!}}\right)}^{\frac{1}{\sum_{i=1}^{m}k_{i}}}},a_{2c-2c{\left(1-{\left(\prod_{\sigma \in E_{m}}\left(1-\prod_{i=1}^{m}{\left(1-{\left(\frac{I_{\sigma (i)}}{2c}\right)}^{mw_{i}}\right)}^{k_{i}}\right)\right)}^{\frac{1}{m!}}\right)}^{\frac{1}{\sum_{i=1}^{m}k_{i}}}},a_{2c-2c{\left(1-{\left(\prod_{\sigma \in E_{m}}\left(1-\prod_{i=1}^{m}{\left(1-{\left(\frac{F_{\sigma (i)}}{2c}\right)}^{mw_{i}}\right)}^{k_{i}}\right)\right)}^{\frac{1}{m!}}\right)}^{\frac{1}{\sum_{i=1}^{m}k_{i}}}}\right) \end{array}.$$ (8)

**Proof.**

Based on Definition 4, $mw_{i}\alpha_{\sigma (i)}=(a_{2c-2c{\left(1-\frac{T_{\sigma (i)}}{2c}\right)}^{mw_{i}}},a_{2c{\left(\frac{I_{\sigma (i)}}{2c}\right)}^{mw_{i}}},a_{2c{\left(\frac{F_{\sigma (i)}}{2c}\right)}^{mw_{i}}})$ and

${\left(mw_{i}\alpha_{\sigma (i)}\right)}^{k_{i}}=(a_{2c{\left(1-{\left(1-\frac{T_{\sigma (i)}}{2c}\right)}^{mw_{i}}\right)}^{k_{i}}},a_{2c-2c{\left(1-{\left(\frac{I_{\sigma (i)}}{2c}\right)}^{mw_{i}}\right)}^{k_{i}}},a_{2c-2c{\left(1-{\left(\frac{F_{\sigma (i)}}{2c}\right)}^{mw_{i}}\right)}^{k_{i}}})$, then

$$\begin{array}{l} \prod_{i=1}^{m}{\left(mw_{i}\alpha_{\sigma (i)}\right)}^{k_{i}}=(a_{2c\prod_{i=1}^{m}{\left(1-{\left(1-\frac{T_{\sigma (i)}}{2c}\right)}^{mw_{i}}\right)}^{k_{i}}},a_{2c-2c\prod_{i=1}^{m}{\left(1-{\left(\frac{I_{\sigma (i)}}{2c}\right)}^{mw_{i}}\right)}^{k_{i}}},a_{2c-2c\prod_{i=1}^{m}{\left(1-{\left(\frac{F_{\sigma (i)}}{2c}\right)}^{mw_{i}}\right)}^{k_{i}}})\;\Rightarrow \\ \sum_{\sigma \in E_{m}}\prod_{i=1}^{m}{\left(mw_{i}\alpha_{\sigma (i)}\right)}^{k_{i}}= \end{array}$$

$$\left(a_{2c-2c\prod_{\sigma \in E_{m}}\left(1-\prod_{i=1}^{m}{\left(1-{\left(1-\frac{T_{\sigma (i)}}{2c}\right)}^{mw_{i}}\right)}^{k_{i}}\right)},a_{2c\prod_{\sigma \in E_{m}}\left(1-\prod_{i=1}^{m}{\left(1-{\left(\frac{I_{\sigma (i)}}{2c}\right)}^{mw_{i}}\right)}^{k_{i}}\right)},a_{2c\prod_{\sigma \in E_{m}}\left(1-\prod_{i=1}^{m}{\left(1-{\left(\frac{F_{\sigma (i)}}{2c}\right)}^{mw_{i}}\right)}^{k_{i}}\right)}\right)$$

$$\Rightarrow \frac{1}{m!}\sum_{\sigma \in E_{m}}\prod_{i=1}^{m}{\left(mw_{i}\alpha_{\sigma (i)}\right)}^{k_{i}}=$$

$$\left(a_{2c-2c{\left(\prod_{\sigma \in E_{m}}\left(1-\prod_{i=1}^{m}{\left(1-{\left(1-\frac{T_{\sigma (i)}}{2c}\right)}^{mw_{i}}\right)}^{k_{i}}\right)\right)}^{\frac{1}{m!}}},a_{2c{\left(\prod_{\sigma \in E_{m}}\left(1-\prod_{i=1}^{m}{\left(1-{\left(\frac{I_{\sigma (i)}}{2c}\right)}^{mw_{i}}\right)}^{k_{i}}\right)\right)}^{\frac{1}{m!}}},a_{2c{\left(\prod_{\sigma \in E_{m}}\left(1-\prod_{i=1}^{m}{\left(1-{\left(\frac{F_{\sigma (i)}}{2c}\right)}^{mw_{i}}\right)}^{k_{i}}\right)\right)}^{\frac{1}{m!}}}\right)$$

$$\Rightarrow {\left(\frac{1}{m!}\sum_{\sigma \in E_{m}}\prod_{i=1}^{m}{\left(mw_{i}\alpha_{\sigma (i)}\right)}^{k_{i}}\right)}^{\frac{1}{\sum_{i=1}^{m}k_{i}}}=$$

$$\left(a_{2c{\left(1-{\left(\prod_{\sigma \in E_{m}}\left(1-\prod_{i=1}^{m}{\left(1-{\left(1-\frac{T_{\sigma (i)}}{2c}\right)}^{mw_{i}}\right)}^{k_{i}}\right)\right)}^{\frac{1}{m!}}\right)}^{\frac{1}{\sum_{i=1}^{m}k_{i}}}},a_{2c-2c{\left(1-{\left(\prod_{\sigma \in E_{m}}\left(1-\prod_{i=1}^{m}{\left(1-{\left(\frac{I_{\sigma (i)}}{2c}\right)}^{mw_{i}}\right)}^{k_{i}}\right)\right)}^{\frac{1}{m!}}\right)}^{\frac{1}{\sum_{i=1}^{m}k_{i}}}},a_{2c-2c{\left(1-{\left(\prod_{\sigma \in E_{m}}\left(1-\prod_{i=1}^{m}{\left(1-{\left(\frac{F_{\sigma (i)}}{2c}\right)}^{mw_{i}}\right)}^{k_{i}}\right)\right)}^{\frac{1}{m!}}\right)}^{\frac{1}{\sum_{i=1}^{m}k_{i}}}}\right)$$

Thus, *LNPMM*^*K*^(*α*_1_,*α*_2_,…,*α*_*m*_) =

$$(a_{2c{\left(1-{\left(\prod_{\sigma \in E_{m}}\left(1-\prod_{i=1}^{m}{\left(1-{\left(1-\frac{T_{\sigma (i)}}{2c}\right)}^{mw_{i}}\right)}^{k_{i}}\right)\right)}^{\frac{1}{m!}}\right)}^{\frac{1}{\sum_{i=1}^{m}k_{i}}}},a_{2c-2c{\left(1-{\left(\prod_{\sigma \in E_{m}}\left(1-\prod_{i=1}^{m}{\left(1-{\left(\frac{I_{\sigma (i)}}{2c}\right)}^{mw_{i}}\right)}^{k_{i}}\right)\right)}^{\frac{1}{m!}}\right)}^{\frac{1}{\sum_{i=1}^{m}k_{i}}}},a_{2c-2c{\left(1-{\left(\prod_{\sigma \in E_{m}}\left(1-\prod_{i=1}^{m}{\left(1-{\left(\frac{F_{\sigma (i)}}{2c}\right)}^{mw_{i}}\right)}^{k_{i}}\right)\right)}^{\frac{1}{m!}}\right)}^{\frac{1}{\sum_{i=1}^{m}k_{i}}}}).$$

**Example 1.** Assume *α*_1_ = (*a*_6_,*a*_3_,*a*_1_), *α*_2_ = (*a*_4_,*a*_2_,*a*_3_), *α*_3_ = (*a*_5_,*a*_4_,*a*_1_) and *α*_4_ = (*a*_3_,*a*_1_,*a*_2_) are four LNNs, and *K* = (1,1,1,1), on the basis of Eq (8), their aggregated value is *gα*_1_ = (*a*_4.35_,*a*_2.68_,*a*_1.84_).

**Property 1.** (Idempotency) Assume $\alpha_{i}=(a_{T_{i}},a_{I_{i}},a_{F_{i}})$ (*i* = 1,2,…,*m*) is a collection of LNNs, and *α*_*i*_ = *α*_*j*_ = *α* = (*a*_*T*_,*a*_*I*_,*a*_*F*_) (*i*,*j* = 1,2,…,*m*) is true, then *LNPMM*^*K*^(*α*_1_,*α*_2_,…,*α*_*m*_) = *α*.

**Proof.**

As *α*_*i*_ = *α*_*j*_ = *α* = (*a*_*T*_,*a*_*I*_,*a*_*F*_), then based on Eq (3), *L*(*α*_*i*_,*α*_*j*_) = 0 ⇒ *G*(*α*_*i*_,*α*_*j*_) = 1−*L*(*α*_*i*_,*α*_*j*_) = 1. Hence, $F(\alpha_{i})=\sum_{j=1,j\neq i}^{m}G(\alpha_{i},\alpha_{j})=m-1$ ⇒ $w_{i}=\frac{\left(1+F(\alpha_{\sigma (i)})\right)}{\sum_{j=1}^{m}\left(1+F(\alpha_{j})\right)}=\frac{1}{m}$.

In addition, by using Eq (8), *LNPMM*^*K*^(*α*_1_,*α*_2_,…,*α*_*m*_) = *LNPMM*^*K*^(*α*,*α*,…,*α*)

$$=\left(a_{2c{\left(1-{\left(\prod_{\sigma \in E_{m}}\left(1-\prod_{i=1}^{m}{\left(1-(1-\frac{T}{2c})\right)}^{k_{i}}\right)\right)}^{\frac{1}{m!}}\right)}^{\frac{1}{\sum_{i=1}^{m}k_{i}}}},a_{2c-2c{\left(1-{\left(\prod_{\sigma \in E_{m}}\left(1-\prod_{i=1}^{m}{\left(1-(\frac{I}{2c})\right)}^{k_{i}}\right)\right)}^{\frac{1}{m!}}\right)}^{\frac{1}{\sum_{i=1}^{m}k_{i}}}},a_{2c-2c{\left(1-{\left(\prod_{\sigma \in E_{m}}\left(1-\prod_{i=1}^{m}{\left(1-(\frac{F}{2c})\right)}^{k_{i}}\right)\right)}^{\frac{1}{m!}}\right)}^{\frac{1}{\sum_{i=1}^{m}k_{i}}}}\right)$$

$$=\left(a_{2c{\left(1-{\left(\prod_{\sigma \in E_{m}}\left(1-{\left(1-(1-\frac{T}{2c})\right)}^{\sum_{i=1}^{m}k_{i}}\right)\right)}^{\frac{1}{m!}}\right)}^{\frac{1}{\sum_{i=1}^{m}k_{i}}}},a_{2c-2c{\left(1-{\left(\prod_{\sigma \in E_{m}}\left(1-{\left(1-(\frac{I}{2c})\right)}^{\sum_{i=1}^{m}k_{i}}\right)\right)}^{\frac{1}{m!}}\right)}^{\frac{1}{\sum_{i=1}^{m}k_{i}}}},a_{2c-2c{\left(1-{\left(\prod_{\sigma \in E_{m}}\left(1-{\left(1-(\frac{F}{2c})\right)}^{\sum_{i=1}^{m}k_{i}}\right)\right)}^{\frac{1}{m!}}\right)}^{\frac{1}{\sum_{i=1}^{m}k_{i}}}}\right)$$

$$=\left(a_{2c{\left(1-{\left(\prod_{\sigma \in E_{m}}\left(1-{\left(\frac{T}{2c}\right)}^{\sum_{i=1}^{m}k_{i}}\right)\right)}^{\frac{1}{m!}}\right)}^{\frac{1}{\sum_{i=1}^{m}k_{i}}}},a_{2c-2c{\left(1-{\left(\prod_{\sigma \in E_{m}}\left(1-{\left(1-\frac{I}{2c}\right)}^{\sum_{i=1}^{m}k_{i}}\right)\right)}^{\frac{1}{m!}}\right)}^{\frac{1}{\sum_{i=1}^{m}k_{i}}}},a_{2c-2c{\left(1-{\left(\prod_{\sigma \in E_{m}}\left(1-{\left(1-\frac{F}{2c}\right)}^{\sum_{i=1}^{m}k_{i}}\right)\right)}^{\frac{1}{m!}}\right)}^{\frac{1}{\sum_{i=1}^{m}k_{i}}}}\right)$$

$$=\left(a_{2c{\left(1-{\left({\left(1-{\left(\frac{T}{2c}\right)}^{\sum_{i=1}^{m}k_{i}}\right)}^{m!}\right)}^{\frac{1}{m!}}\right)}^{\frac{1}{\sum_{i=1}^{m}k_{i}}}},a_{2c-2c{\left(1-{\left({\left(1-{\left(1-\frac{I}{2c}\right)}^{\sum_{i=1}^{m}k_{i}}\right)}^{m!}\right)}^{\frac{1}{m!}}\right)}^{\frac{1}{\sum_{i=1}^{m}k_{i}}}},a_{2c-2c{\left(1-{\left({\left(1-{\left(1-\frac{F}{2c}\right)}^{\sum_{i=1}^{m}k_{i}}\right)}^{m!}\right)}^{\frac{1}{m!}}\right)}^{\frac{1}{\sum_{i=1}^{m}k_{i}}}}\right)$$

$$=\left(a_{2c{\left(1-\left(1-{\left(\frac{T}{2c}\right)}^{\sum_{i=1}^{m}k_{i}}\right)\right)}^{\frac{1}{\sum_{i=1}^{m}k_{i}}}},a_{2c-2c{\left(1-\left(1-{\left(1-\frac{I}{2c}\right)}^{\sum_{i=1}^{m}k_{i}}\right)\right)}^{\frac{1}{\sum_{i=1}^{m}k_{i}}}},a_{2c-2c{\left(1-\left(1-{\left(1-\frac{F}{2c}\right)}^{\sum_{i=1}^{m}k_{i}}\right)\right)}^{\frac{1}{\sum_{i=1}^{m}k_{i}}}}\right)$$

$$=\left(a_{2c{\left({\left(\frac{T}{2c}\right)}^{\sum_{i=1}^{m}k_{i}}\right)}^{\frac{1}{\sum_{i=1}^{m}k_{i}}}},a_{2c-2c{\left({\left(1-\frac{I}{2c}\right)}^{\sum_{i=1}^{m}k_{i}}\right)}^{\frac{1}{\sum_{i=1}^{m}k_{i}}}},a_{2c-2c{\left({\left(1-\frac{F}{2c}\right)}^{\sum_{i=1}^{m}k_{i}}\right)}^{\frac{1}{\sum_{i=1}^{m}k_{i}}}}\right)$$

$$=(a_{2c\cdot \frac{T}{2c}},a_{2c-2c\cdot (1-\frac{I}{2c})},a_{2c-2c\cdot (1-\frac{F}{2c})})=(a_{T},a_{I},a_{F})=\alpha .$$

**Property 2.** (Boundedness) If $\alpha_{i}=(a_{T_{i}},a_{I_{i}},a_{F_{i}})$ (*i* = 1,2,…,*m*) is a set of LNNs, *α*^+^ = max{*α*_1_,*α*_2_,…,*α*_*m*_} = (*a*_*T*+_,*a*_*I*+_,*a*_*F*+_) and *α*^−^ = min{*α*_1_,*α*_2_,…,*α*_*m*_} = (*a*_*T*−_,*a*_*I*−_,*a*_*F*−_), then

$$LNPMM^{K}\overset{m}{\overset{︷}{\left(\alpha^{-},\alpha^{-},\ldots ,\alpha^{-}\right)}}\leq LNPMM^{K}(\alpha_{1},\alpha_{2},\ldots ,\alpha_{m})\leq LNPMM^{K}\overset{m}{\overset{︷}{\left(\alpha^{+},\alpha^{+},\ldots ,\alpha^{+}\right)}}.$$

**Proof.**

According to Eq (8), *LNPMM*^*K*^(*α*_1_,*α*_2_,…,*α*_*m*_) =

$$\left(a_{2c{\left(1-{\left(\prod_{\sigma \in E_{m}}\left(1-\prod_{i=1}^{m}{\left(1-{\left(1-\frac{T_{\sigma (i)}}{2c}\right)}^{mw_{i}}\right)}^{k_{i}}\right)\right)}^{\frac{1}{m!}}\right)}^{\frac{1}{\sum_{i=1}^{m}k_{i}}}},a_{2c-2c{\left(1-{\left(\prod_{\sigma \in E_{m}}\left(1-\prod_{i=1}^{m}{\left(1-{\left(\frac{I_{\sigma (i)}}{2c}\right)}^{mw_{i}}\right)}^{k_{i}}\right)\right)}^{\frac{1}{m!}}\right)}^{\frac{1}{\sum_{i=1}^{m}k_{i}}}},a_{2c-2c{\left(1-{\left(\prod_{\sigma \in E_{m}}\left(1-\prod_{i=1}^{m}{\left(1-{\left(\frac{F_{\sigma (i)}}{2c}\right)}^{mw_{i}}\right)}^{k_{i}}\right)\right)}^{\frac{1}{m!}}\right)}^{\frac{1}{\sum_{i=1}^{m}k_{i}}}}\right)$$

$$As\;(a_{{\left(1-\frac{T_{\sigma (i)}}{2c}\right)}^{mw_{i}}},a_{1-{\left(\frac{I_{\sigma (i)}}{2c}\right)}^{mw_{i}}},a_{1-{\left(\frac{F_{\sigma (i)}}{2c}\right)}^{mw_{i}}})\leq (a_{{\left(1-\frac{T+}{2c}\right)}^{mw_{i}}},a_{1-{\left(\frac{I+}{2c}\right)}^{mw_{i}}\prod_{i=1}^{m}{\left(\right)}^{k_{i}}},a_{1-{\left(\frac{F}{2c}\right)}^{mw_{i}}})$$

$$\begin{array}{l} \Rightarrow \\ \left(a_{\prod_{i=1}^{m}{\left(1-{\left(1-\frac{T_{\sigma (i)}}{2c}\right)}^{mw_{i}}\right)}^{k_{i}}},a_{1-\prod_{i=1}^{m}{\left(1-{\left(\frac{I_{\sigma (i)}}{2c}\right)}^{mw_{i}}\right)}^{k_{i}}},a_{1-\prod_{i=1}^{m}{\left(1-{\left(\frac{F_{\sigma (i)}}{2c}\right)}^{mw_{i}}\right)}^{k_{i}}})\leq (a_{\prod_{i=1}^{m}{\left(1-{\left(1-\frac{T+}{2c}\right)}^{mw_{i}}\right)}^{k_{i}}},a_{1-\prod_{i=1}^{m}{\left(1-{\left(\frac{I+}{2c}\right)}^{mw_{i}}\right)}^{k_{i}}},a_{1-\prod_{i=1}^{m}{\left(1-{\left(\frac{F}{2c}\right)}^{mw_{i}}\right)}^{k_{i}}}\right) \end{array}$$

$$\Rightarrow \;\left(a_{{\left(\prod_{\sigma \in E_{m}}\left(1-\prod_{i=1}^{m}{\left(1-{\left(1-\frac{T_{\sigma (i)}}{2c}\right)}^{mw_{i}}\right)}^{k_{i}}\right)\right)}^{\frac{1}{m!}}},a_{1-{\left(\prod_{\sigma \in E_{m}}\left(1-\prod_{i=1}^{m}{\left(1-{\left(\frac{I_{\sigma (i)}}{2c}\right)}^{mw_{i}}\right)}^{k_{i}}\right)\right)}^{\frac{1}{m!}}},a_{1-{\left(\prod_{\sigma \in E_{m}}\left(1-\prod_{i=1}^{m}{\left(1-{\left(\frac{F_{\sigma (i)}}{2c}\right)}^{mw_{i}}\right)}^{k_{i}}\right)\right)}^{\frac{1}{m!}}}\right)$$

$$\leq (a_{{\left(\prod_{\sigma \in E_{m}}\left(1-\prod_{i=1}^{m}{\left(1-{\left(1-\frac{T+}{2c}\right)}^{mw_{i}}\right)}^{k_{i}}\right)\right)}^{\frac{1}{m!}}},a_{1-{\left(\prod_{\sigma \in E_{m}}\left(1-\prod_{i=1}^{m}{\left(1-{\left(\frac{I+}{2c}\right)}^{mw_{i}}\right)}^{k_{i}}\right)\right)}^{\frac{1}{m!}}},a_{1-{\left(\prod_{\sigma \in E_{m}}\left(1-\prod_{i=1}^{m}{\left(1-{\left(\frac{F+}{2c}\right)}^{mw_{i}}\right)}^{k_{i}}\right)\right)}^{\frac{1}{m!}}})$$

$$\Rightarrow \;\left(a_{2c{\left(1-{\left(\prod_{\sigma \in E_{m}}\left(1-\prod_{i=1}^{m}{\left(1-{\left(1-\frac{T_{\sigma (i)}}{2c}\right)}^{mw_{i}}\right)}^{k_{i}}\right)\right)}^{\frac{1}{m!}}\right)}^{\frac{1}{\sum_{i=1}^{m}k_{i}}}},a_{2c-2c{\left(1-{\left(\prod_{\sigma \in E_{m}}\left(1-\prod_{i=1}^{m}{\left(1-{\left(\frac{I_{\sigma (i)}}{2c}\right)}^{mw_{i}}\right)}^{k_{i}}\right)\right)}^{\frac{1}{m!}}\right)}^{\frac{1}{\sum_{i=1}^{m}k_{i}}}},a_{2c-2c{\left(1-{\left(\prod_{\sigma \in E_{m}}\left(1-\prod_{i=1}^{m}{\left(1-{\left(\frac{F_{\sigma (i)}}{2c}\right)}^{mw_{i}}\right)}^{k_{i}}\right)\right)}^{\frac{1}{m!}}\right)}^{\frac{1}{\sum_{i=1}^{m}k_{i}}}}\right)$$

$$\leq (a_{2c{\left(1-{\left(\prod_{\sigma \in E_{m}}\left(1-\prod_{i=1}^{m}{\left(1-{\left(1-\frac{T+}{2c}\right)}^{mw_{i}}\right)}^{k_{i}}\right)\right)}^{\frac{1}{m!}}\right)}^{\frac{1}{\sum_{i=1}^{m}k_{i}}}},a_{2c-2c{\left(1-{\left(\prod_{\sigma \in E_{m}}\left(1-\prod_{i=1}^{m}{\left(1-{\left(\frac{I+}{2c}\right)}^{mw_{i}}\right)}^{k_{i}}\right)\right)}^{\frac{1}{m!}}\right)}^{\frac{1}{\sum_{i=1}^{m}k_{i}}}},a_{2c-2c{\left(1-{\left(\prod_{\sigma \in E_{m}}\left(1-\prod_{i=1}^{m}{\left(1-{\left(\frac{F+}{2c}\right)}^{mw_{i}}\right)}^{k_{i}}\right)\right)}^{\frac{1}{m!}}\right)}^{\frac{1}{\sum_{i=1}^{m}k_{i}}}})$$

As *α*^+^ = max{*α*_1_,*α*_2_,…,*α*_*m*_} = (*a*_*T*+_,*a*_*I*+_,*a*_*F*+_), according to Eq (8),

$$\left(a_{2c{\left(1-{\left(\prod_{\sigma \in E_{m}}\left(1-\prod_{i=1}^{m}{\left(1-{\left(1-\frac{T+}{2c}\right)}^{mw_{i}}\right)}^{k_{i}}\right)\right)}^{\frac{1}{m!}}\right)}^{\frac{1}{\sum_{i=1}^{m}k_{i}}}},a_{2c-2c{\left(1-{\left(\prod_{\sigma \in E_{m}}\left(1-\prod_{i=1}^{m}{\left(1-{\left(\frac{I+}{2c}\right)}^{mw_{i}}\right)}^{k_{i}}\right)\right)}^{\frac{1}{m!}}\right)}^{\frac{1}{\sum_{i=1}^{m}k_{i}}}},a_{2c-2c{\left(1-{\left(\prod_{\sigma \in E_{m}}\left(1-\prod_{i=1}^{m}{\left(1-{\left(\frac{F+}{2c}\right)}^{mw_{i}}\right)}^{k_{i}}\right)\right)}^{\frac{1}{m!}}\right)}^{\frac{1}{\sum_{i=1}^{m}k_{i}}}}\right)$$

= $LNPMM^{K}\overset{m}{\overset{︷}{\left(\alpha^{+},\alpha^{+},\ldots ,\alpha^{+}\right)}}$. Hence, $LNPMM^{K}(\alpha_{1},\alpha_{2},\ldots ,\alpha_{m})\leq LNPMM^{K}\overset{m}{\overset{︷}{\left(\alpha^{+},\alpha^{+},\ldots ,\alpha^{+}\right)}}$.

Similarly, it is true that $LNPMM^{K}(\alpha_{1},\alpha_{2},\ldots ,\alpha_{m})\geq LNPMM^{K}\overset{m}{\overset{︷}{\left(\alpha^{-},\alpha^{-},\ldots ,\alpha^{-}\right)}}$.

Thus, $LNPMM^{K}\overset{m}{\overset{︷}{\left(\alpha^{-},\alpha^{-},\ldots ,\alpha^{-}\right)}}\leq LNPMM^{K}(\alpha_{1},\alpha_{2},\ldots ,\alpha_{m})\leq LNPMM^{K}\overset{m}{\overset{︷}{\left(\alpha^{+},\alpha^{+},\ldots ,\alpha^{+}\right)}}$.

In the following, some special cases of LNPMM operators are explored:

**Special case 1:** When *K* = (1,0,…,0), the LNPMM operator is degraded into the linguistic neutrosphic power average operator, denoted as:

$$LNPMM^{\left(1,0,\ldots ,0\right)}(\alpha_{1},\alpha_{2},\ldots ,\alpha_{m})=\sum_{i=1}^{m}\left(w_{i}\alpha_{i}\right)=\sum_{i=1}^{m}\left(\frac{\left(1+F(\alpha_{\sigma (i)})\right)}{\sum_{j=1}^{m}\left(1+F(\alpha_{j})\right)}\alpha_{i}\right).$$ (9)

**Special case 2:**

When $K=(\frac{1}{m},\frac{1}{m},\ldots ,\frac{1}{m})$, the LNPMM operator is degenerated into the linguistic neutrosphic power geometric operator, denoted as:

$$LNPMM^{\left(\frac{1}{m},\frac{1}{m},\ldots ,\frac{1}{m}\right)}(\alpha_{1},\alpha_{2},\ldots ,\alpha_{m})=\prod_{i=1}^{m}{\left(\alpha_{i}\right)}^{w_{i}}=\prod_{i=1}^{m}{\left(\alpha_{i}\right)}^{\frac{\left(1+F(\alpha_{\sigma (i)})\right)}{\sum_{j=1}^{m}\left(1+F(\alpha_{j})\right)}}.$$ (10)

**Special case 3:**

When *K* = (1,1,0,0,…,0), the LNPMM operator is degenerated into the linguistic neutrosphic power Bonferroni mean operator, denoted as:

$$\begin{array}{l} \;LNPMM^{\left(1,1,0,0,\ldots ,0\right)}(\alpha_{1},\alpha_{2},\ldots ,\alpha_{m})=\\ (a_{2c{\left(1-{\left(\prod_{i,j=1,i\neq j}^{m}\left(1-\left(1-{\left(1-\frac{T_{i}}{2c}\right)}^{w_{i}}\right)\left(1-{\left(1-\frac{T_{j}}{2c}\right)}^{w_{j}}\right)\right)\right)}^{\frac{1}{m(m-1)}}\right)}^{\frac{1}{2}}},a_{2c-2c{\left(1-{\left(\prod_{i,j=1,i\neq j}^{m}\left(1-\left(1-{\left(\frac{I_{i}}{2c}\right)}^{w_{i}}\right)\left(1-{\left(\frac{I_{j}}{2c}\right)}^{w_{j}}\right)\right)\right)}^{\frac{1}{m(m-1)}}\right)}^{\frac{1}{2}}},a_{2c-2c{\left(1-{\left(\prod_{i,j=1,i\neq j}^{m}\left(1-\left(1-{\left(\frac{F_{i}}{2c}\right)}^{w_{i}}\right)\left(1-{\left(\frac{F_{j}}{2c}\right)}^{w_{j}}\right)\right)\right)}^{\frac{1}{m(m-1)}}\right)}^{\frac{1}{2}}}). \end{array}$$ (11)

**Special case 4:**

When $K=(\overset{l}{\overset{︷}{1,1,\ldots ,1}},\overset{m-l}{\overset{︷}{0,0,\ldots ,0}})$, the LNPMM operator is degenerated into the linguistic neutrosphic power Maclaurin symmetric mean operator, denoted as:

$$\begin{array}{l} LNPMM^{\left(\overset{l}{\overset{︷}{1,1,\ldots ,1}},\overset{m-l}{\overset{︷}{0,0,\ldots ,0}}\right)}(\alpha_{1},\alpha_{2},\ldots ,\alpha_{m})=\\ (a_{2c{\left(1-\prod_{1\leq i_{1}<\ldots <i_{l}\leq m}{\left(1-\prod_{j=1}^{l}\left(1-{\left(1-\frac{T_{i_{j}}}{2c}\right)}^{w_{i_{j}}}\right)\right)}^{\frac{1}{C_{m}^{l}}}\right)}^{\frac{1}{l}}},a_{2c-2c{\left(1-\prod_{1\leq i_{1}<\ldots <i_{l}\leq m}{\left(1-\prod_{j=1}^{l}\left(1-{\left(\frac{I_{i_{j}}}{2c}\right)}^{w_{i_{j}}}\right)\right)}^{\frac{1}{C_{m}^{l}}}\right)}^{\frac{1}{l}}},a_{2c-2c{\left(1-\prod_{1\leq i_{1}<\ldots <i_{l}\leq m}{\left(1-\prod_{j=1}^{l}\left(1-{\left(\frac{F_{i_{j}}}{2c}\right)}^{w_{i_{j}}}\right)\right)}^{\frac{1}{C_{m}^{l}}}\right)}^{\frac{1}{l}}}). \end{array}$$ (12)

**Special case 5:**

When *K* = (1,1,…,1), the LNPMM operator is degraded into the linguistic neutrosphic power geometric average operator, denoted as:

$$LNPMM^{\left(1,1,\ldots ,1\right)}(\alpha_{1},\alpha_{2},\ldots ,\alpha_{m})=\prod_{i=1}^{m}{\left(w_{i}\alpha_{i}\right)}^{\frac{1}{m}}=\prod_{i=1}^{m}{\left(\frac{\left(1+F(\alpha_{\sigma (i)})\right)}{\sum_{j=1}^{m}\left(1+F(\alpha_{j})\right)}\alpha_{i}\right)}^{\frac{1}{m}}.$$ (13)

Unlike the linguistic neutrosphic power geometric average operator in Special case 1, the linguistic neutrosphic power geometric average operator emphasizes the equilibrium of arguments and the coordination (instead of complementarity) among individuals.

### Weighted linguistic neutrosophic power Muirhead mean operator

Clearly, the weights of LNNs are not under considerations in the LNPMM operators. Thus, the WLNPMM operators are proposed in this subsection, so that the weights of LNNs can be contained. In other words, if the weights of criteria need to be considered, DMs should choose the WLNPMM operators, otherwise the LNPMM operators can be selected.

**Definition 11.** Given several LNNs *α*_*i*_ (*i* = 1,2,…,*m*), *K* = (*k*_1_,*k*_2_,…,*k*_*m*_)∈*R*^*m*^ is a vector of parameters, *σ*(*i*) is any permutation of (*i* = 1,2,…,*m*), *E*_*m*_ is a set of all possible permutations, $F(\alpha_{i})=\sum_{j=1,j\neq i}^{m}G(\alpha_{i},\alpha_{j})$, and 0≤*G*(*α*_*i*_,*α*_*j*_) = 1−*L*(*α*_*i*_,*α*_*j*_)≤1 is the support for *α*_*i*_ and *α*_*j*_, $w_{i}=\frac{\left(1+F(\alpha_{\sigma (i)})\right)}{\sum_{j=1}^{m}\left(1+F(\alpha_{j})\right)}\in [0,1]$, $\sum_{i=1}^{m}w_{i}=1$, 0≤*ϖ*_*i*_≤1 is the weight value, and *w*_1_+*w*_2_+⋯+*w*_*m*_ = 1, then WLNPMM operator is

$$WLNPMM^{K}(\alpha_{1},\alpha_{2},\ldots ,\alpha_{m})={\left(\frac{1}{m!}\sum_{\sigma \in E_{m}}\prod_{i=1}^{m}{\left(\frac{mw_{\vartheta (i)}\varpi_{\sigma (i)}}{\sum_{j=1}^{m}w_{j}\varpi_{j}}\alpha_{\sigma (i)}\right)}^{k_{i}}\right)}^{\frac{1}{\sum_{i=1}^{m}k_{i}}}.$$ (14)

**Theorem 2.** If there are several LNNs $\alpha_{i}=(a_{T_{i}},a_{I_{i}},a_{F_{i}})$ (*i* = 1,2,…,*m*), then the aggregated value based on Eq (14) is a LNN, where

$$\begin{array}{l} WLNPMM^{K}(\alpha_{1},\alpha_{2},\ldots ,\alpha_{m})=\\ (a_{2c{\left(1-{\left(\prod_{\sigma \in E_{m}}\left(1-\prod_{i=1}^{m}{\left(1-{\left(1-\frac{T_{\sigma (i)}}{2c}\right)}^{\frac{mw_{\vartheta (i)}\varpi_{\sigma (i)}}{\sum_{j=1}^{m}w_{j}\varpi_{j}}}\right)}^{k_{i}}\right)\right)}^{\frac{1}{m!}}\right)}^{\frac{1}{\sum_{i=1}^{m}k_{i}}}},a_{2c-2c{\left(1-{\left(\prod_{\sigma \in E_{m}}\left(1-\prod_{i=1}^{m}{\left(1-{\left(\frac{I_{\sigma (i)}}{2c}\right)}^{\frac{mw_{\vartheta (i)}\varpi_{\sigma (i)}}{\sum_{j=1}^{m}w_{j}\varpi_{j}}}\right)}^{k_{i}}\right)\right)}^{\frac{1}{m!}}\right)}^{\frac{1}{\sum_{i=1}^{m}k_{i}}}},a_{2c-2c{\left(1-{\left(\prod_{\sigma \in E_{m}}\left(1-\prod_{i=1}^{m}{\left(1-{\left(\frac{F_{\sigma (i)}}{2c}\right)}^{\frac{mw_{\vartheta (i)}\varpi_{\sigma (i)}}{\sum_{j=1}^{m}w_{j}\varpi_{j}}}\right)}^{k_{i}}\right)\right)}^{\frac{1}{m!}}\right)}^{\frac{1}{\sum_{i=1}^{m}k_{i}}}}). \end{array}$$ (15)

**Example 2.** Suppose *α*_1_ = (*a*_6_,*a*_3_,*a*_1_), *α*_2_ = (*a*_4_,*a*_2_,*a*_3_), *α*_3_ = (*a*_5_,*a*_4_,*a*_1_) and *α*_4_ = (*a*_3_,*a*_1_,*a*_2_) are four LNNs, *K* = (1,1,1,1) and *ϖ*_1_ = *ϖ*_2_ = *ϖ*_3_ = *ϖ*_4_ = 1/4, based on Eq (15), their aggregated value is *gα*_2_ = (*a*_4.36_,*a*_2.69_,*a*_1.84_).

## New linguistic neutrosophic evaluation approach

In this section, a new approach is presented with the WLNPMM operator to address mine safety evaluation problems within linguistic neutrosophic circumstances.

### Problem description

Given that there are *p* mines {*R*_1_,*R*_2_,⋯,*R*_*p*_}, and DMs are required to assess the safety of these mines under *q* criteria {*S*_1_,*S*_2_,⋯,*S*_*q*_}, so that the safest mine can be selected. *ϖ*_*j*_ is the corresponding weight value of criterion *S*_*j*_ (*j* = 1,2,…,*q*), where *ϖ*_*j*_∈[0,1] and $\sum_{j=1}^{q}\varpi_{j}=1$. Besides, experts decide to express their preference by means of LNNs. Hence, a linguistic neutrosophic evaluation matrix is constructed, denoted as *U* = (*α*_*ij*_)_*p*×*q*_, where $\alpha_{ij}=(a_{T_{ij}},a_{I_{ij}},a_{F_{ij}})$ is the linguistic neutrosophic evaluation information of mine *R*_*i*_ (*i* = 1,2,⋯,*p*) against criterion *S*_*j*_ (*j* = 1,2,⋯,*q*).

### Decision making process

The decision making procedures for coping with mine safety evaluation problems are:

Step 1: Normalize the original assessment matrix.

Generally, when both benefit and cost criteria exist in the matrix simultaneously, the cost criteria need to be converted to the benefit one for convenience. The transformation rule is

$$\alpha_{ij}^{N}=\{\begin{array}{l} (a_{T_{ij}},a_{I_{ij}},a_{F_{ij}})\;for\;benefit\;criteria\;S_{j}\\ (a_{F_{ij}},a_{I_{ij}},a_{T_{ij}})\;for\;\cos t\;criteria\;S_{j} \end{array}.$$ (16)

Consequently, the normalized decision making matrix is $U^{N}={\left(\alpha_{ij}^{N}\right)}_{p\times q}$.

Step 2: Obtain the power weight values.

The power weight value *w*_*ij*_ of the corresponding LNN $\alpha_{ij}^{N}$ can be computed with

$$G(\alpha_{ij}^{N},\alpha_{ir}^{N})=1-L(\alpha_{ij}^{N},\alpha_{ir}^{N})(j,r=1,2,\ldots ,q;r\neq j)(j,r=1,2,\ldots ,q;r\neq j),$$ (17)

$$F(\alpha_{ij}^{N})=\sum_{r=1,r\neq j}^{q}G(\alpha_{ij}^{N},\alpha_{ir}^{N}),$$ (18)

$$w_{ij}=\frac{\left(1+F(\alpha_{ij}^{N})\right)}{\sum_{r=1}^{q}\left(1+F(\alpha_{ir}^{N})\right)}.$$ (19)

Step 3: Acquire the comprehensive assessment values.

Based on the WLNPMM operator defined in subsection 3.2, the overall assessment value is

$$V_{i}=WLNPMM^{K}(\alpha_{i1}^{N},\alpha_{i2}^{N},\ldots ,\alpha_{iq}^{N}).$$ (20)

Step 4: Compute the score function or accuracy function.

According to Eq (1), the score function *B*(*V*_*i*_) of each mine is computed. When two score function values are the same, the accuracy function values of them should be computed based on Eq (2).

Step 5: Determine the safest mine.

In accordance with Eqs (1) and (2), the safest mine R* can be obtained.

## Case study

In this section, an case of safety assessment for gold mines is illustrated to justify the practicability of our approach.

### Project profile

Laizhou city is located in Shandong Province of China. It is an important gold production base, where distributes numerous gold mines. Nevertheless, near-to surface mineral resources in most of these mines are gradually becoming depleted with the accelerating rate of mining in recent decades. Exploiting deep mineral resources has become unavoidable. However, the situation of safety production is becoming more and more serious because of the higher ground stress, temperature, and water pressure in deep mining areas. To protect the lives and property of workers effectively, it is essential to conduct a safety evaluation for these mines firstly.

To understand the mine safety status, the local mine safety supervision bureau intends to evaluate the safety conditions of four typical gold mines (denoted as *R*_1_, *R*_2_, *R*_3_ and *R*_4_) in this area recently.

### Evaluation criteria

Identifying the criteria is the first step for the mine safety evaluation. Based on the concrete characteristics of mines and some literature [3,6,7], four criteria are selected after thorough investigations: the human factor, environmental conditions, technological equipment, and management quality (denoted as *S*_1_, *S*_2_, *S*_3_ and *S*_4_). The details of these criteria are described in **Table 1**.

**Table 1:** Details of evaluation criteria for mine safety.

Evaluation criteria	Benefit/Cost	Descriptions
Human factor *S*_1_	Benefit	It refers to the personal protection, emergency training, violation, and total mining experience.
Environmental conditions *S*_2_	Benefit	It refers to the geological feature, dust content, temperature, and humidity.
Technological equipment *S*_3_	Benefit	It refers to the mining mechanization, ventilation, dust-proof, fire-fighting, drainage, and transport equipment.
Management quality *S*_4_	Benefit	It refers to the monitoring, defective design, safety culture, rules and regulations.

### Determining the safest mine

Suppose the importance degrees of these four criteria are equal, that is, *ϖ*_1_ = *ϖ*_2_ = *ϖ*_3_ = *ϖ*_4_ = 1/4. Considering the fuzziness of human cognitions, LNNs are suggested to describe these four qualitative evaluation indexes for reserving initial evaluation information as much as possible. A decision making group, which contain ten experts, is planned to make evaluations. The used linguistic term set is

$$A=\{a_{0}=very\;low,\;a_{1}=low,\;a_{2}=a\;little\;low,\;a_{3}=medium,\;a_{4}=a\;little\;high,\;a_{5}=high,\;a_{6}=very\;high\}.$$

After mutual discussions, the initial evaluation information is obtained with LNNs in **Table 2**.

**Table 2:** Original assessment matrix *U*.

*U*	*S*_1_	*S*_2_	*S*_3_	*S*_4_
*R*_1_	(*a*_5_,*a*_2_,*a*_1_)	(*a*_6_,*a*_3_,*a*_2_)	(*a*_3_,*a*_1_,*a*_3_)	(*a*_4_,*a*_2_,*a*_3_)
*R*_2_	(*a*_4_,*a*_2_,*a*_0_)	(*a*_5_,*a*_1_,*a*_3_)	(*a*_3_,*a*_4_,*a*_2_)	(*a*_3_,*a*_1_,*a*_2_)
*R*_3_	(*a*_6_,*a*_3_,*a*_1_)	(*a*_4_,*a*_2_,*a*_3_)	(*a*_5_,*a*_4_,*a*_1_)	(*a*_3_,*a*_1_,*a*_2_)
*R*_4_	(*a*_4_,*a*_1_,*a*_2_)	(*a*_3_,*a*_4_,*a*_2_)	(*a*_4_,*a*_2_,*a*_3_)	(*a*_6_,*a*_2_,*a*_3_)

Then, the new methodology is used to pick out a gold mine with best safety conditions. The detailed procedures are described in the following.

Step 1: Normalize the original evaluation matrix.

As all criteria are benefit, they don’t need to be transformed, then the normalized matrix is still *U*^*N*^ = *U*.

Step 2: Obtain the power weight values.

By using Eq (17), the supports for two LNNs are calculated (let *λ* = 1) (See the second to fourth columns in **Tables 3–6**).

**Table 3:** Values of $G(\alpha_{ij}^{N},\alpha_{ir}^{N})$, $F(\alpha_{ij}^{N})$ and *w*_*ij*_ (*i* = 1).

$G(\alpha_{ij}^{N},\alpha_{ir}^{N})$	$\alpha_{11}^{N}$	$\alpha_{12}^{N}$	$\alpha_{13}^{N}$	$\alpha_{14}^{N}$	$F(\alpha_{1j}^{N})$	*w*_1*j*_
$\alpha_{11}^{N}$	--	0.833	0.722	0.833	2.388	0.252
$\alpha_{12}^{N}$	0.833	--	0.667	0.778	2.278	0.244
$\alpha_{13}^{N}$	0.722	0.667	--	0.889	2.278	0.244
$\alpha_{14}^{N}$	0.833	0.778	0.889	--	2.500	0.260

**Table 4:** Values of $G(\alpha_{ij}^{N},\alpha_{ir}^{N})$, $F(\alpha_{ij}^{N})$ and *w*_*ij*_ (*i* = 2).

$G(\alpha_{ij}^{N},\alpha_{ir}^{N})$	$\alpha_{21}^{N}$	$\alpha_{22}^{N}$	$\alpha_{23}^{N}$	$\alpha_{24}^{N}$	$F(\alpha_{2j}^{N})$	*w*_2*j*_
$\alpha_{21}^{N}$	--	0.722	0.722	0.778	2.222	0.246
$\alpha_{22}^{N}$	0.722	--	0.667	0.833	2.222	0.246
$\alpha_{23}^{N}$	0.722	0.667	--	0.833	2.222	0.246
$\alpha_{24}^{N}$	0.778	0.833	0.833	--	2.444	0.262

**Table 5:** Values of $G(\alpha_{ij}^{N},\alpha_{ir}^{N}),\;F(\alpha_{ij}^{N})$ and *w*_*ij*_ (*i* = 3).

$G(\alpha_{ij}^{N},\alpha_{ir}^{N})$	$\alpha_{31}^{N}$	$\alpha_{32}^{N}$	$\alpha_{33}^{N}$	$\alpha_{34}^{N}$	$F(\alpha_{3j}^{N})$	*w*_3*j*_
$\alpha_{31}^{N}$	--	0.722	0.889	0.667	2.278	0.252
$\alpha_{32}^{N}$	0.722	--	0.722	0.833	2.277	0.252
$\alpha_{33}^{N}$	0.889	0.722	--	0.667	2.278	0.252
$\alpha_{34}^{N}$	0.667	0.833	0.667	--	2.167	0.244

**Table 6:** Values of $G(\alpha_{ij}^{N},\alpha_{ir}^{N})$, $F(\alpha_{ij}^{N})$ and *w*_*ij*_ (*i* =4).

$G(\alpha_{ij}^{N},\alpha_{ir}^{N})$	$\alpha_{41}^{N}$	$\alpha_{42}^{N}$	$\alpha_{43}^{N}$	$\alpha_{44}^{N}$	$F(\alpha_{4j}^{N})$	*w*_4*j*_
$\alpha_{41}^{N}$	--	0.778	0.889	0.778	2.445	0.254
$\alpha_{42}^{N}$	0.778	--	0.778	0.667	2.223	0.238
$\alpha_{43}^{N}$	0.889	0.778	--	0.889	2.556	0.262
$\alpha_{44}^{N}$	0.778	0.667	0.889	--	2.334	0.246

Then, based on Eq (18), the values of $F(\alpha_{ij}^{N})$ are computed (See the sixth columns in **Tables 3–6**).

Thereafter, the power weight values are calculated on the basis of Eq (19) (See the last columns in **Tables 3–6**).

Step 3: Acquire the comprehensive evaluation results.

Based on the WLNPMM defined in subsection 3.2, the comprehensive evaluation results are (Without loss of generality, we assume *K* = (1,1,1,1)): $V_{1}=WLNPMM^{K}(\alpha_{11}^{N},\alpha_{12}^{N},\alpha_{13}^{N},\alpha_{14}^{N})=(a_{4.36},a_{2.06},a_{2.34})$, *V*_2_ = (*a*_3.67_,*a*_2.25_,*a*_1.88_), *V*_3_ = (*a*_4.35_,*a*_2.68_,*a*_1.84_) and *V*_4_ = (*a*_4.12_,*a*_2.46_,*a*_2.53_).

Step 4: Compute the score or accuracy values.

By using Eq (1), the score function of each mine is computed as: *B*(*V*_1_)≈0.664, *B*(*V*_2_)≈0.641, *B*(*V*_3_)≈0.657 and *B*(*V*_4_)≈0.618.

Step 5: Determine the optimal alternative.

As *B*(*V*_1_)> *B*(*V*_3_)> *B*(*V*_2_)> *B*(*V*_4_), then the ranking order of all mines is *R*_1_≻*R*_3_≻*R*_2_≻*R*_4_.

## Discussions

The impact of parameters is discussed, and the strengths of the proposed aggregation operators are justified in this section.

### Sensitivity analyses

In this subsection, the impacts of the parameter vector *K* = (*k*_1_,*k*_2_,*k*_3_,*k*_4_) in the LNPMM operator are analyzed. Ranking results are obtained when dissimilar values are assigned to *K* (See **Table 7**).

**Table 7:** Ranking orders under dissimilar *K* values.

Parameter vector *K*	Score function value	Ranking order	Best alternative R*
*K* = (1,0,0,0)	*B*(*V*_1_)≈0.782, *B*(*V*_2_)≈0.792, *B*(*V*_3_)≈0.790, *B*(*V*_4_)≈0.754.	*R*_2_≻*R*_3_≻*R*_1_≻*R*_4_	*R*_2_
*K* = (1,1,0,0)	*B*(*V*_1_)≈0.526, *B*(*V*_2_)≈0.512, *B*(*V*_3_)≈0.527, *B*(*V*_4_)≈0.486.	*R*_3_≻*R*_1_≻*R*_2_≻*R*_4_	*R*_3_
*K* = (1,1,1,0)	*B*(*V*_1_)≈0.673, *B*(*V*_2_)≈0.655, *B*(*V*_3_)≈0.669, *B*(*V*_4_)≈0.629.	*R*_1_≻*R*_3_≻*R*_2_≻*R*_4_	*R*_1_
*K* = (1,1,1,1)	*B*(*V*_1_)≈0.664, *B*(*V*_2_)≈0.641, *B*(*V*_3_)≈0.657, *B*(*V*_4_)≈0.618.	*R*_1_≻*R*_3_≻*R*_2_≻*R*_4_	*R*_1_
*K* = (1/4,1/4,1/4,1/4)	*B*(*V*_1_)≈0.664, *B*(*V*_2_)≈0.642, *B*(*V*_3_)≈0.657, *B*(*V*_4_)≈0.621.	*R*_1_≻*R*_3_≻*R*_2_≻*R*_4_	*R*_1_

From **Table 7**, it is clear that dissimilar rankings are derived with different *K* values. When the criteria are independent with each other, i.e., *K* = (1,0,0,0), the score values of alternatives are greatest, and the best one is *R*_2_. When the relations between two LNNs are captured, the best alternative is changed as *R*_3_. However, the ranking result is stable at *R*_1_≻*R*_3_≻*R*_2_≻*R*_4_ when more interrelations among linguistic neutroshophic criteria values are reflected. In other words, the best alternative is *R*_1_ in most cases (i.e., *K* = (1,1,1,0), *K* = (1,1,1,1) and *K* = (1/4,1/4,1/4,1/4)). Therefore, the proposed method is robust in some extent. Meanwhile, the choice of parameter can reflect DMs’ risk preference and increase the flexibility of this method. That is, when the DM is optimistic, she/he can choose a smaller *K* (*K* = (1,0,0,0) or *K* = (1,1,0,0)), to obtain more flexibility; On the contrary, when the DM is pessimistic, he/she may choose a larger *K* to retain more stability.

### Validation of the proposed approach

In this subsection, an example from literature [57] is used to verify the feasibility of our method firstly. Then, comparison analyses with several literature [35,40,41,43] are made to show the strengths of our approach.

Part 1: Validation with a same example

In this part, our method is adopted to solve the problem in literature [57]. The dataset can be seen in [57] and the detailed process is:

Step 1: Normalize the original evaluation matrix.

The normalized original evaluation matrix is the same with that in [57].

Step 2: Obtain the power weight values.

Based on Eq (16)–(18), the power weight values are calculated as: *w*_1*j*_ = {0.254,0.254,0.246,0.246}, *w*_2*j*_ = {0.246,0.254,0.254,0.246}, *w*_3*j*_ = {0.256,0.256,0.232,0.256} and *w*_4*j*_ = {0.254,0.254,0.238,0.254}.

Step 3: Acquire the comprehensive evaluation results.

Based on the WLNPMM defined in subsection 3.2, the comprehensive evaluation results are (Let *K* = (1,1,1,1)): *e*_1_ = (*a*_4.37_,*a*_2.33_,*a*_1.32_), *e*_2_ = (*a*_4.36_,*a*_2.34_,*a*_1.32_), *e*_3_ = (*a*_4.35_,*a*_2.33_,*a*_1.33_) and *e*_4_ = (*a*_4.36_,*a*_2.33_,*a*_1.32_).

Step 4: Compute the score or accuracy values.

By using Eq (1), the score function of each mine is computed as: *U*(*e*_1_)≈0.7067, *U*(*e*_2_)≈0.7056, *U*(*e*_3_)≈0.7050 and *U*(*e*_4_)≈0.7061.

Step 5: Determine the optimal alternative.

As *U*(*e*_1_)> *U*(*e*_4_)> *U*(*e*_2_)> *U*(*e*_3_), then the ranking order is *α*_1_≻*α*_4_≻*α*_2_≻*α*_3_.

As the ranking result is the same with that in [57], it demonstrates the feasibility of our method to some extent.

Part 2: Comparison of ranking results

At first, the ranking orders of alternatives in Section 5 with different approaches are obtained, as listed in **Table 8**.

**Table 8:** Rankings with different approaches.

Approach	Ranking basis	Value	Ranking order
LNWAM [35]	Score function value	*B*(*V*_1_)≈0.782, *B*(*V*_2_)≈0.792, *B*(*V*_3_)≈0.790, *B*(*V*_4_)≈0.753.	*R*_2_≻*R*_3_≻*R*_1_≻*R*_4_
LNWGM [35]	Score function value	*B*(*V*_1_)≈0.664, *B*(*V*_2_)≈0.641, *B*(*V*_3_)≈0.657, *B*(*V*_4_)≈0.619.	*R*_1_≻*R*_3_≻*R*_2_≻*R*_4_
Cosine-based [40]	Cosine measure	*cm*(*V*_1_)≈0.958, *cm*(*V*_2_)≈0.946, *cm*(*V*_3_)≈0.959, *cm*(*V*_4_)≈0.933.	*R*_3_≻*R*_1_≻*R*_2_≻*R*_4_
TOPSIS [41]	Distance measure	*dm*(*V*_1_)≈0.375, *dm*(*V*_2_)≈0.346, *dm*(*V*_3_)≈0.375, *dm*(*V*_4_)≈0.337.	*R*_1_~*R*_3_≻*R*_2_≻*R*_4_
MULTIMOORA [43]	Ratio system, reference point and multiplicative model	Rank 1: *R*_4_≻*R*_1_≻*R*_3_≻*R*_2_ Rank 2: *R*_4_≻*R*_1_≻*R*_2_≻*R*_3_ Rank 3: *R*_4_≻*R*_2_≻*R*_3_≻*R*_1_	*R*_4_≻*R*_1_≻*R*_2_≻*R*_3_
The proposed approach	Score function value	*B*(*V*_1_)≈0.664, *B*(*V*_2_)≈0.641, *B*(*V*_3_)≈0.657, *B*(*V*_4_)≈0.618.	*R*_1_≻*R*_3_≻*R*_2_≻*R*_4_

As dissimilar rankings exist in **Table 8**, the best ranking among them needs to be determined to certify the effectiveness of the proposed approach. For this purpose, the technique in literature [69] is suggested.

Step 1: Compute the number of times for alternatives under various ranks in **Table 9**. For example, it can be seen that *R*_2_ ranks No.1 once and No.3 five times.

**Table 9:** Number of times for mines under different ranks.

Mines	Ranks			
	1	2	3	4
*R*_1_	3	2	1	
*R*_2_	1		5	
*R*_3_	2	3		1
*R*_4_	1			5

Step 2: Smooth the alternatives in terms of ranking distribution, as shown in **Table 10**.

**Table 10:** Smoothing of mines (Π_*is*_).

Mines	Ranks			
	1	2	3	4
*R*_1_	3	5	6	6
*R*_2_	1	1	6	6
*R*_3_	2	5	5	6
*R*_4_	1	1	1	6

Step 3: Establish a programming model with several constraints as follows:

$$\begin{array}{l} Max\;\Psi =\sum_{i=1}^{4}\sum_{s=1}^{4}(\Pi_{is}\cdot \frac{4^{2}}{s}\cdot \Phi_{is})\\ s.t.\{\begin{array}{c} \sum_{i=1}^{4}\Phi_{is}=1,\;s=1,2,3,4\\ \sum_{s=1}^{4}\Phi_{is}=1,\;i=1,2,3,4\\ \Phi_{is}=0\;or\;\Phi_{is}=1,\;i,s=1,2,3,4 \end{array}. \end{array}$$ (21)

After addressing this model, the optimum ranking is *R*_1_≻*R*_3_≻*R*_2_≻*R*_4_.

**Fig 1:**
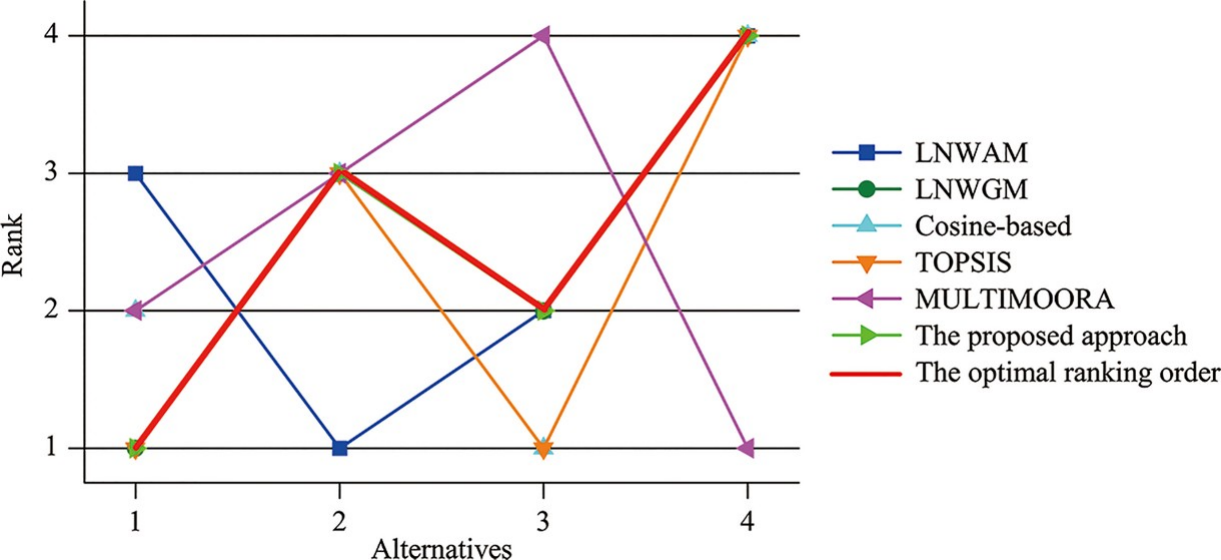
Ranking orders with different methods.

The optimal ranking and other rankings in **Table 8** are portrayed, which can be seen in **Fig 1**. Obviously, same rankings are derived with Model (21) and our method. It demonstrates that the proposed method is preponderant in disposing such issues where extreme values exist or the interrelations among criteria should be captured.

Part 3: Further comparisons

As indicated in **Table 8**, various techniques have been adopted to resolve evaluation issues under linguistic neutrosophic environment. They are based on dissimilar ranking bases and have distinctive characteristics.

(1) Compared with existent methods based on other aggregation operators

The LNWAM and LNWGM operators in literature [35] are two basic linguistic neutrosophic mean operators. However, both of them don’t take the mutual relationships of LNNs into account. Instead, our approach can reflect the interrelations among several inputs. Even though the approach in literature [43,54,55,56,57] also considers the relationship of arguments, it neglects the influences of unreasonable information. On the other hand, just the correlations between two inputs can be captured in [43,54,55], while the relations among more than two arguments are reflected in [56,57]. Compared with them, the proposed method can describe the relationships among any number of inputs through the adjustment of parameter vector. Furthermore, the idea of power weighting is borrowed in our method, so that the impacts of some irrational values can be diminished. In this sense, the advised approach is more influential and supple.

(2) Compared with other existent decision making approaches

The approach proposed by Shi and Ye in literature [40] is based on cosine measure. That is, the cosine measures of pairwise LNNs need to be calculated before ranking alternatives. Similarly, the distance measures of pairwise LNNs are required to be compute when the extended TOPSIS in literature [41] is adopted. Clearly, a lot of additional calculations are produced in these methods. More seriously, the approach in [43] is based on three modes, which makes it complicated. In addition, a satisfactory ranking may be not derived in some cases, especially when three induced ranks are contradictory. In contrast, the proposed method in this study is on the basis of aggregation of LNNs under each alternative. In other words, the score functions of aggregated values can directly obtain the final rankings. Compared with them [40,41,43], our method is simple and has less computing work.

### Summary of advantages

In summary, the highlights of our approach are:

1. Due to the vagueness of DMs, the fuzzy evaluation data are expressed by LNNs. In LNNs, three independent linguistic membership functions are included. In this case, not only the consistent and inconsistent information, but also the hesitant degrees of DMs can be conveniently and fully depicted.
2. The constructed framework is based on the WLNPMM, so that the superiority of PA and MM operators can be exploited. That is, the proposed method can capture the relationships among any number of inputs with the MM operators. At the same time, our method can avoid negative influence of bad data with the PA operators.
3. Many existent aggregation operators (e.g., the arithmetic/geometric/Bonferroni mean operators) can be regarded as the special cases of MM operators. Thus, the proposed method, which combined MM operators with PA operators and LNNs, is more general. Besides, the alterable parameter vector makes our method more flexible as it can alter with the change of the number of inputs whose relations can be reflected.

## Conclusions

In this study, the LNPMM operator and WLNPMM operator were explored to aggregate linguistic neutrosophic information. The highlight of these operators is that they can exert the advantages of LNNs, PA and MM operators. Some main properties and special cases of them were revealed as well. Then, the new decision making methodology with these operators was adopted to evaluate the safety of mines under linguistic neutrosophic environment. The strengths include: The correlations among criteria can be reflected and the negative impacts of anomalous values on ranking orders can be diminished in the evaluation process. The sensitivity analysis certified that our method is flexible because it contains a changeable parameter vector. Meanwhile, the comparison analyses with other methods showed that our method is robust and efficient when solving complex decision making problems under linguistic neutrosophic conditions.

The limitation of this study is that the way of determining criteria weight values is not discussed. Because plenty of objective and subjective weight determination models have been developed in existent literature, maybe they can be directly used or duly modified according to the characteristics of issues. Second, the proposed method in this study may be adopted to settle linguistic neutrosophic decision making issues in other fields. Third, maybe our method can combined with other extensions of neutrosophic sets (such as the plithogenic set [70,71] and neutrosophic linguistic overset/underset/offset [72] according to the real conditions. In addition, the proposed method can be applied into other fields, and new applications (especially applications in industry) with knowledge of neutrosophic and plithogenic sets and logic are worthy to be researched in the future.
